# Bilateral giant juvenile fibroadenoma of breasts

**DOI:** 10.4103/0971-9261.55156

**Published:** 2009

**Authors:** Madhumita Mukhopadhyay, Rishavdeb Patra, Sima Mondal, Asit Ghosh, A. K. Ray

**Affiliations:** Department of Pathology, IPGME&R and SSKM Hospital, Kolkata-20, India; 1 Department of Pediatric Surgery, IPGME&R and SSKM Hospital, Kolkata-20, India

**Keywords:** Breast tumor, fine needle aspiration cytology, fibroadenoma, phyllodes tumor

## Abstract

An 11-year-old girl with rapidly enlarging bilateral breast lumps is reported. It was diagnosed as a case of juvenile fibroadenoma following fine needle aspiration cytology and confirmed on histopathological examination of the excised specimens.

## INTRODUCTION

Bilaterally symmetrical giant juvenile fibroadenomas are very rare in prepubertal girls.[1,2] The lesions are well circumscribed, lobulated and can cause ulceration of the overlying skin because of rapid growth.[2]

## CASE REPORT

**Figure 1:**
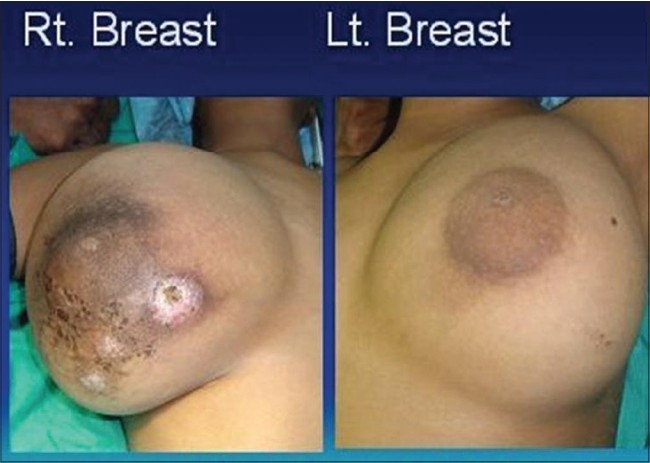
Bilateral breast tumors

An 11-year-old premenarche girl presented with bilateral, rapidly increasing breast lumps for two months [Figure 1]. There was a history of dull ache in the breasts. There was no history of trauma, nipple discharge, fever, anorexia or weight loss. There was no significant family history. Clinical examination revealed mobile, slightly tender lobulated lumps. The right breast lump measured 22 cm × 20cm and left breast lump measured 18cm × 16cm. Both the lumps were solitary, well circumscribed and not fixed to the deeper structures. There was an area of skin ulceration overlying the lump on the right side [Figure 1]. There was no axillary lymphadenopathy. Routine hematological and biochemical examinations were within normal limits. Chest X Ray was normal. Ultrasonography of both the breasts showed heterogeneous parenchymal pattern.

**Figure 2:**
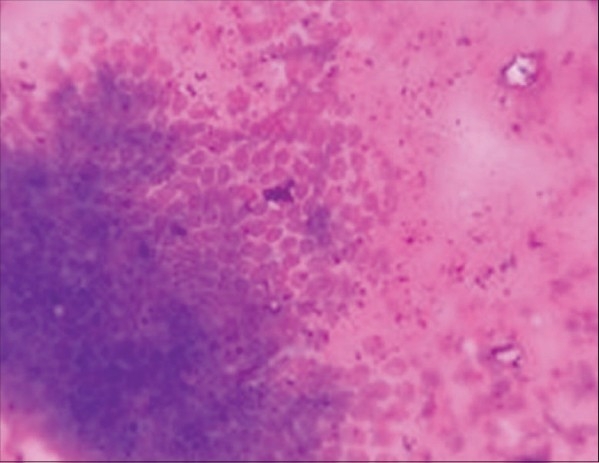
FNAC from breast lumps (MGG stain; 40 × 10) show aggregate of cohesive epithelial cells

**Figure 3:**
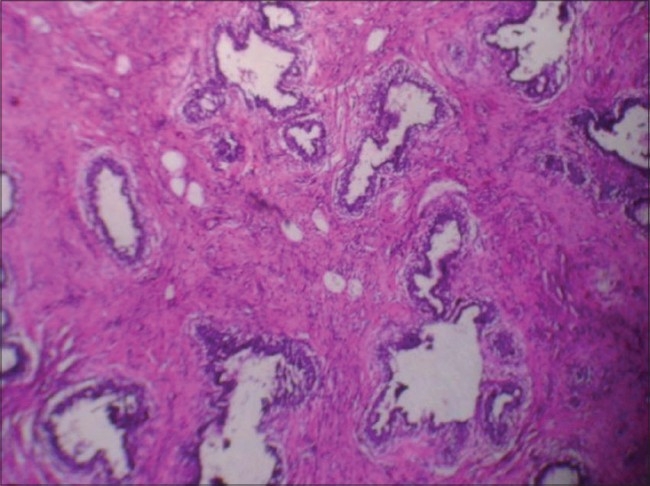
Histopathology (H and E Stain; Photomicrograph- 10 × 10) shows increased cellularity of Stromal and parenchymal component. no periductal concentrates of cells

FNAC showed aggregates of cohesive epithelial cells [Figure 2]. A few bipolar nuclei and bare nuclei were also noted. There was no evidence of malignancy. Excision of bilateral breast lumps conserving the normal breast tissues along with nipple and areola was done. Cut surface of the lumps were solid, grayish white and bulging with whorled appeareance. Hematoxylin and eosin (H and E) stain showed [Figure 3] increased cellularity of stromal and parenchymal component. Stromal cellularity was increased but there was no focal periductal concentrate of cells; a characteristic of phyllodes tumor. The patient is on regular follow-up and doing well at six months follow-up. We do not plan prosthesis or augmentation now.

## DISCUSSION

Giant juvenile fibroadenoma in the prepubertal age group is almost always benign and should be treated with breast conserving surgery.[3,4] Juvenile giant fibroadenoma should be distinguished from phyllodes tumor. The distinction is very important because the former should be treated with excision of lumps and preservation of surrounding normal breast tissue. In phyllodes tumor, a rim of normal tissue should be included in the excised lump. This distinct type of fibroadenoma that tends to occur in adolescents shows hypercellularity of glands and stroma.[5] A plethora of names exist to designate the lesion such as age related term Juvenile fibroadenoma and size related like giant or massive fibroadenoma.[6] Giant fibroadenomas constitute about 4% of all fibroadenomas in the breast.[6] The occurrence of fibroadenoma which is large and at the same time hyper cellular should be differentiated from virginal hypertrophy and phyllodes tumor. Sometimes it is difficult to distinguish clinically juvenile giant fibroadenoma from phyllodes tumor.[6] Although malignant tumors of the breast are rare in this age group, two per cent of all primary malignant breast lesions occur under the age of 25 years in females.[7]

Various other conditions like lipoma, hamartoma, cysts should be kept in the list of differential diagnosis.[8] Most of the times, physical examination, imaging like ultrasonography of breasts, mammography and magnetic resonance imaging fail to make diagnosis.[6] The stromal hypercellularity should be evaluated more carefully in terms of presence of atypical cell. It is also rare for phyllodes tumor to occur in young patients.[9]

Giant juvenile fibroadenoma simultaneously occurring in both the breasts is rare. Isolated case reports are available in the English literature.[10,11] Sometimes it is difficult to diagnose by FNAC. In our patient, the diagnosis was made clinically, substantiated by FNAC and confirmed by histopathology.[12,13] Fortunately, majority of these tumors can be removed completely by simple mastectomy, preserving the nipple and areola, as was done in our patient.[14] Giant juvenile fibroadenoma may recur after complete excision and the chance of recurrence becomes less after third decade.[15]
